# Examples of shifting development pathways: lessons on how to enable broader, deeper, and faster climate action

**DOI:** 10.1007/s44168-022-00026-1

**Published:** 2022-12-15

**Authors:** Harald Winkler, Franck Lecocq, Hans Lofgren, Maria Virginia Vilariño, Sivan Kartha, Joana Portugal-Pereira

**Affiliations:** 1 Policy Research in International Services and Manufacturing, School of Economics, and associate at African Climate and Development Initiative, University of Cape Town, Cape Town, South Africa; 2 Centre International de Recherche sur l’Environnement et le Développement (CIRED), Université Paris-Saclay, AgroParisTech, CNRS, Ecole des Ponts ParisTech, CIRAD, EHESS, Nogent-sur-Marne, 94130, France; 3 Independent Researcher, Swarthmore, USA; 4 Consejo Empresario Argentino para el Desarrollo Sostenible, Buenos Aires, Argentina; 5 Stockholm Environment Institute, 11 Curtis Avenue, Somerville, MA 02144-1224 USA; 6 CENERGIA/PPE/COPPE, Universidade Federal Do Rio de Janeiro (UFRJ), Rio de Janeiro, Brazil; 7 IDMEC, Instituto Superior Técnico, University of Lisbon, Lisbon, Portugal; 8 Centre for Environmental Policy, Imperial College London, London, UK

**Keywords:** Shifting development pathways towards sustainability, Enabling conditions, Sustainable development, Mitigation, Policy packages, Decarbonisation, Climate-change policy

## Abstract

To respond to the climate crisis, we need to accelerate system transformations at a pace, scale, and breadth not seen before. This means that it is urgent to shift development pathways towards net zero greenhouse gas emissions, even while progressing towards other sustainable development objectives. This paper argues that accelerated mitigation can not only benefit from policies that are outside the domain of conventional emission-focused mitigation policies but *require* such policies. We refer to this process as *shifting development pathways towards sustainability*. Here, we explore what enabling conditions make such shifts possible. We develop a framework to select examples of shifts — in realms such as educational access, housing access, fiscal arrangements, and institutional reform. We analyse them against key enablers. Our findings suggest that countries could learn from what has worked elsewhere, though context matters. Some enablers are more widely applicable, including finance, long-term vision, and focus on sustainable development objectives. Multiple enablers, integrated policy packages, and involvement of a broad range of actors help achieve multiple objectives. Some enablers may yield results in the near term, while others take time to yield results. Based on our analysis, we suggest that climate mitigation requires an “all of economy, all of society” approach.

**Graphical Abstract:**

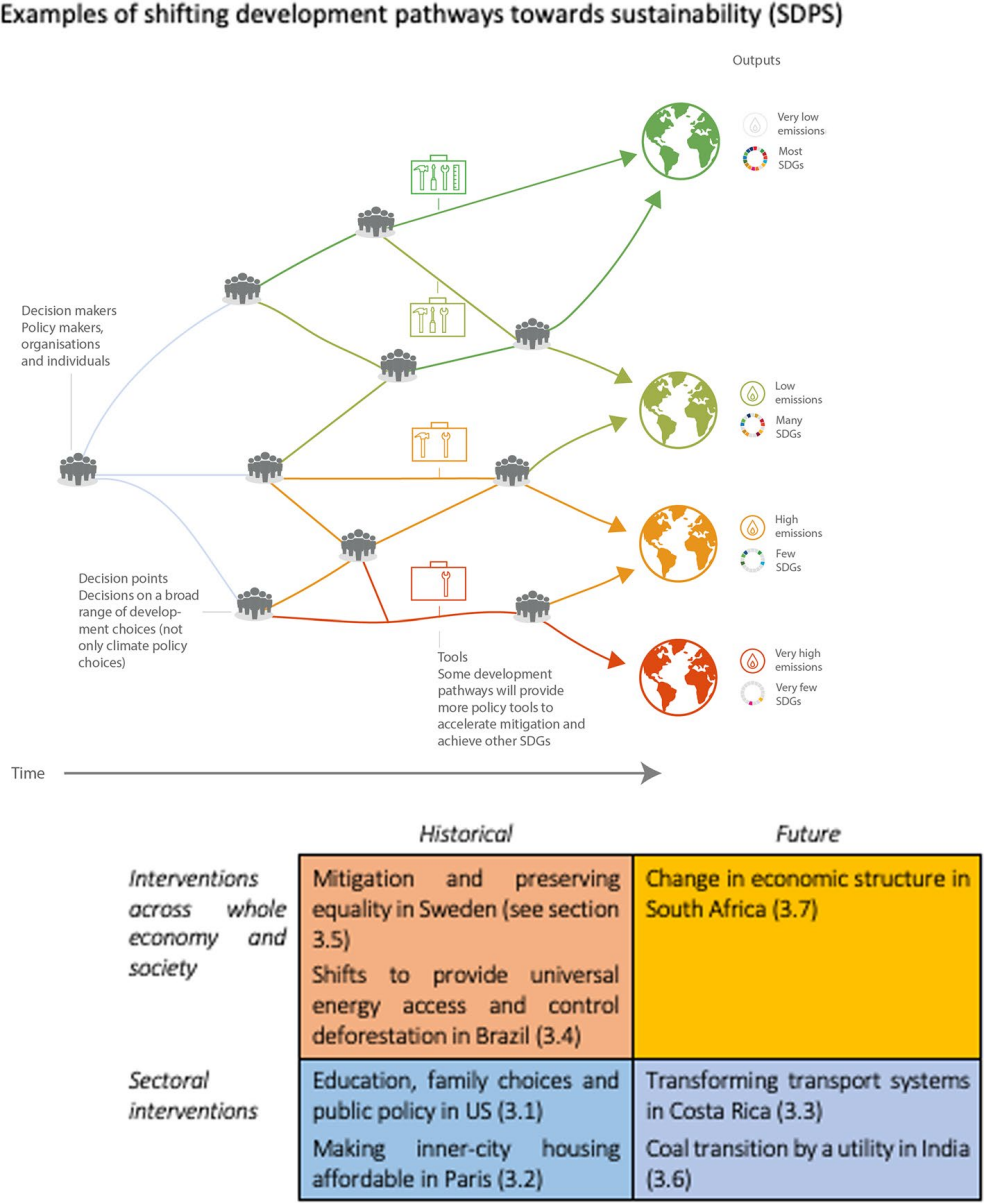

## Introduction

Why is it urgent to shift development pathways? Because, to address the climate crisis, we need to accelerate low-carbon transitions at a pace, scale, and breadth not seen before. The Intergovernmental Panel on Climate Change (IPCC) has long provided assessments on how to accelerate mitigation (IPCC 1990, 2018), and yet greenhouse gas (GHG) emissions keep rising to record levels reaching nearly 60 GtCO_2_e in 2020 (Höhne et al. 2023; UNEP 2021). A complementary and broader framing is needed, to shift development paths to towards sustainability (SDPS), and has emerged in the IPCC Working Group III Sixth Assessment Report (AR6) (IPCC 2022b; Lecocq et al. 2022a).

Previous IPCC assessments urged a faster pace of mitigation, i.e. reinforcing the need to implement responses to reduce GHG emissions across all countries and systems. The WG III contribution to AR6 found that efforts embedding mitigation “within the wider development context can increase the pace, depth and breadth of emissions reductions to limit global warming to 2°C or 1.5°C” (IPCC 2022b). The requirements to broaden participation were discussed since 2011 at COP11 in Montreal, yet COP26 in Glasgow 15 years later stated that “warming to 1.5 °C requires rapid, deep and sustained reductions in global greenhouse gas emissions” (UNFCCC 2021). The scientific assessment by WG III in AR6 framed the challenge as “Shifting development pathways to increase sustainability and broaden mitigation options” (IPCC 2022a; Lecocq et al. 2022a).

The SDPS concept, as it emerged in AR6, is designed to highlight that to make a non-incremental, discontinuous changes possible, it is necessary to strengthen the enabling conditions for mitigation and sustainable development. Yet, as a concept, SDPS may seem vague. Here, we make it more concrete by analysing examples of how SDPS has been done and can be made possible. To our knowledge, the paper is the first in the literature that, drawing on a set of cases, analyses how to shift development pathways towards sustainability.

While the concept of SDPS is new, it has deep roots in the literature and assessments. Our paper builds on earlier literature on mainstreaming climate into development (for example Bickersteth et al. 2017; Halsnaes and Shukla 2005), earlier assessment of sustainable development in climate change (IPCC et al. 2001; Pahle et al. 2021; Sathaye et al. 2007), connected with poverty and inequality (Roy et al. 2018). However, earlier IPCC assessments did not explicitly include development pathways as a major focus.

Drawing on a set of selected examples of SDPS, we address the following research questions: what enabling conditions have made these shifts possible? What lessons, applicable in other contexts, can be extracted from successful examples of SDPS? What enablers are context-specific, or even tied to specific interventions, and which are more broadly applicable?

The overall research design is implemented in the following steps. First, we develop working definitions of key terms and outline a framework for selecting examples of SDPS (“Concepts and case study method”). Next, we present seven examples of SDPS (“Examples of how to shift development pathways”) as case studies and assess them against a set of enablers (Assessment of examples of SDPS across enablers). We conclude on lessons learned from examples of SDPS in different contexts and how such shifts can broaden options for mitigation.

## Concepts and case study method

The concept of SDPS emerged for the first time in the assessment of literature by the IPCC’s Working Group III Sixth Assessment Report (AR6) in its summary for policymakers and a cross-chapter box (Lecocq et al. 2022a). As framed in the report, “shifting development pathways towards sustainability offers ways to broaden the range of levers and enablers that a society can use to accelerate mitigation and increases the likelihood of making progress simultaneously on climate action and other development goals” (Pathak et al. 2022). This means that there is growing evidence in the literature should that integrated public policies that focus on choices taken by many actors that shift development pathways can broaden and deepen mitigation action. In this section, we introduce some key concepts used in an empirical analysis of cases of SDPS. Our approach follows an inductive analysis, in which we assessed selected case studies of SDPS in different regions and systems, identified key levers and enablers, and drawn general lessons. While theoretical methods, or deductive analyses, could have been applied, this exploratory study makes no claim to present a theory or explanatory model.

### Introducing concepts

We use some key terms and outline what we mean in working definitions. Development pathways evolve as the result of the countless decisions being made and actions being taken at all levels of societal structure, as well due to the emergent dynamics within and between institutions, cultural norms, technological systems, and other drivers of behavioural change (IPCC 2022a, b: see Glossary). Shifts of development pathways (SDP) introduce the concept that transitions aim at redirecting existing development trends — though the direction is not signalled in SDP. IPCC WG III found that societies may put in place enabling conditions, and we analyse these across several case studies. Here, we are exploring the case where societies seek to make decisions and take actions that influence their future development pathway in ways that advance progress towards sustainability.

In this paper, we address shifting development pathways towards sustainability, an intended shift with a direction to increased sustainability (IPCC 2022a, b: see Glossary). It is also possible that shifts occur without intention, responding, for example to lower prices of renewable energy. In considering enablers and policy packages, we focus our paper on intentional shifts. We argue that it is possible to shift development pathways through policies, examining a set of cases. The UN Agenda 2030 Sustainable Development Goals (SDGs) provide useful lens to assess the multiple dimensions of sustainable development (Nilsson et al. 2016; United Nations General Assembly 2015). Climate action is one of the 17 agreed goals. In our analysis, we examine cases defined around national development objectives, which may or (more likely) may not be explicitly framed in terms of the SDGs.

Various terms could be used to refer to the tools that societies use to induce shifts. We interchangeably use the terms enabling conditions and enablers to refer to such tools (and do not refer to other terms used in the literature: drivers (Lachapelle and Paterson 2013; Le Quéré et al. 2019; Steckel et al. 2015), levers (Winkler et al. 2015), leverage points, or places to intervene in a system (Meadows 1999). We also refer to tools but more as a metaphor to convey that a broader toolbox enables the shift in development pathways to increase sustainability, including mitigation. We choose enabling conditions as the main term, since we intend this paper to be policy relevant and the term is well understood by policymakers. Enablers convey a broader sense of interventions than government policy instruments. The set of enabling conditions is described in “Assessment of examples of SDPS across enablers” and illustrated in Fig. 3 below.

### Case study analysis

The methodology in this paper follows a qualitative approach. Our methods are focused on identifying enabling factors and policies that, as a package, could shift development pathways towards sustainability from specific case studies. We draw information from existing studies (cited in the “Examples of how to shift development pathways”) to assess the cases. This includes both empirical and modelled data and limited to a sector or economy side (as elaborated in “Method of selecting cases of SDPS”). We did not undertake any coding of literature but present cases in “Examples of how to shift development pathways” based on literature review. We assess the cases against enabling conditions (in “Assessment of examples of SDPS across enablers”).

Case studies have long been used in social sciences to draw lessons for public policy (Rose 1991) and used as strategic research methodologies (Noor 2008). Case studies are included in handbooks on qualitative research (Flyvbjerg 2011). Another author suggests that learning lessons and creating policy transparency are important in achieving goals in climate policy (Aldy 2014), and we suggest to climate and other sustainable development goals. The merits and challenges of generalising from case studies are well understood (Tsang 2014). We apply the well-established methods of case studies analysis, and we take care in outlining the limitations of our research findings and basing our findings on the examples selected. We have not pursued a large-n study and make no claim that the cases here are statistically representative.

To our knowledge, the paper is perhaps the first in the literature that, drawing on a set of cases, analyses how to shift development pathways towards sustainability. We refer to the short case studies also as examples, using “cases” and “examples” interchangeably.

### Method of selecting cases of SDPS

Table 1 presents the framework for selecting a set of examples of SDPS. Our method reflects two dimensions of change as proposed by Grübler (1996). A temporal dimension distinguishes between historical examples and future scenarios, while a spatial dimensions refer to economy-wide shifts as distinct from interventions specific to a sector. Note that IPCC reports increasingly refer to sectors also as systems, pointing to interlinkages with other sectors.

**Table 1:** Framework for selection of cases of shifting development paths to increased sustainability

	Historical	Future
**Interventions across whole economy and society**	Mitigation and preserving equality in Sweden (see Mitigation and preserving equality in Sweden section) Shifts to provide universal energy access and control deforestation in Brazil (Shifts to provide universal energy access and control illegal deforestation in Brazil)	Change in economic structure in South Africa (Change in economic structure in South Africa)
**Sectoral interventions**	Education, family choices, and public policy in the USA (Education, family choices and public policy) Making inner-city housing affordable in Paris (Making inner-cities affordable and accessible to low- and middle-income households)	Transforming transport systems in Costa Rica (Transformational change in transport systems in Costa Rica) Coal transition by a utility in India (Coal transition led by a state-owned enterprise in India)

Source: Authors

The case study selection followed the following criteria: (i) including at least one case for each temporal-spatial combination and (ii) geographical coverage. The study presents at least one case for each quadrant, covering the combinations of temporal and spatial dimensions as explained above. We have indicated in brackets after each case in Table 1 in which sub-section the detailed description can be found, and the literature for each is cited there. Secondly, it represents different contexts from all regions of the globe, including countries at various levels of development, as well as different sectors of the economy. As the IPCC indicates, “countries at all stages of economic development seek to improve the well-being of people, and their development priorities reflect different starting points and contexts. Different contexts include social, economic, environmental, cultural, or political conditions, resource endowment, capabilities, international environment, and history” (IPCC 2022b).

Although this is far from being an exhaustive systematic literature review, it allows us to include examples of policy interventions from different contexts in Africa, Asia, Europe, Latin America, and the Caribbean and North America. Without any claim to be representative, we consider all major geographical regions.

Having explained the method for selecting cases, it is worth emphasising that shifts in development pathways may have taken place in the past, enabling ex post analysis. To explore shifts that have not yet occurred, modelling tools are used in the literature for ex ante analysis. We draw on existing cases, for both empirical case and modelled ones. A final methodological point is that economy-wide shifts of development pathway are particularly complex, given interactions across multiple systems and sectors within an economy and society.

## Examples of how to shift development pathways

This section describes seven examples of how to shift development pathways. Each case in the framework for selection (Table 1 below) is developed in a sub-section. We aim to illustrate several practical options to shift development pathways, in ways that both advance development objectives, including reduced GHG emissions, and strengthen the set of available enablers.

### Education, family choices, and public policy

One of the drivers of transportation emissions is urban sprawl, which is itself driven by a range of underlying causes (see IPCC AR6, Chap. 8). One such cause is the wide differences in educational quality offered in different localities in many countries, combined with the strong incentive for households with children to seek housing in localities with higher quality educational options. Thus, choice of schools serves as an important case wherein broader societal developmental choices act as a driver for emissions; in this case, education policy and the resulting household behaviours drive emissions through its impacts on the spatial organisation of society. While we focus here at the household level, families shape individual behaviour through socialisation, and environmental values and practices are transmitted intergenerationally (Litina et al. 2016; Meeusen 2014).

A large literature examining the relationship between housing prices and school quality empirically finds that families will pay a significant premium to gain access to schools that are seen to be higher quality. This pattern has been identified in data relating to different levels of education and in many countries (Machin 2011; Nguyen-Hoang and Yinger 2011). In many areas, the educational options available in different communities are of markedly varied quality and consequently are regularly ranked among the most important determinants of where families with school-age children choose to reside (Batchis 2010). People commute from far away to good schools, adding to transportation demand and thereby emissions. Thus, in these contexts, educational systems that shape where people choose to live contribute to urban sprawl.

Urban sprawl will not be contained without changes in education policy — so that is a mitigation-relevant policy. In the US context, for example, changing education policy would require a change in its funding. If the tax base was not limited to the local community, funding of schools that was less disparate across communities could be achieved. This could reduce a major driver of travel time and/or sprawl and thus also reduce emissions. Additionally, and perhaps even more importantly, education builds capacity in the youth of a country and thus is fundamental to building capacity for mitigation, adaptation, and sustainable development. Educational policy is thus a case in which it is necessary to draw on policy enablers well outside of conventional climate policy in order to achieve important climate-related goals. The IPCC refers to conventional climate policy instruments, “such as emissions taxes or permits, price incentives such as feed-in tariffs for low-carbon electricity generation, and fuel economy standards, and building codes” (IPCC 2022a: section 4.3.1.3), and in its assessment finds that such instruments on their own will not achieve the long-term goals of the Paris Agreement. Conventional climate policies influence only proximate drivers of emissions, whereas SDPS can influence ultimate drivers too (see Raskin et al. 2002).

### Making inner cities affordable and accessible to low- and middle-income households

Exclusionary mechanisms such as decreasing accessibility and affordability of inner-urban neighbourhoods are a major cause of suburbanization of low- to middle-income households in many countries (Hochstenbach and Musterd 2018). When low- and middle-wage jobs (clerks, salesmen, waiters, etc.) do not follow suit, suburbanisation leads to higher demand for transportation (Bento et al. 2005) and higher carbon footprints for households (Jones and Kammen 2014). In fact, evidence suggest that increasing housing prices in metropolitan areas actually push jobs and housing apart, thereby increasing commuting distance (Blumenberg and King 2021; Blumenberg and Wander 2022). Similarly, other studies find significant positive link between housing prices and energy demand (Lampin et al. 2013).

**Fig. 1:**
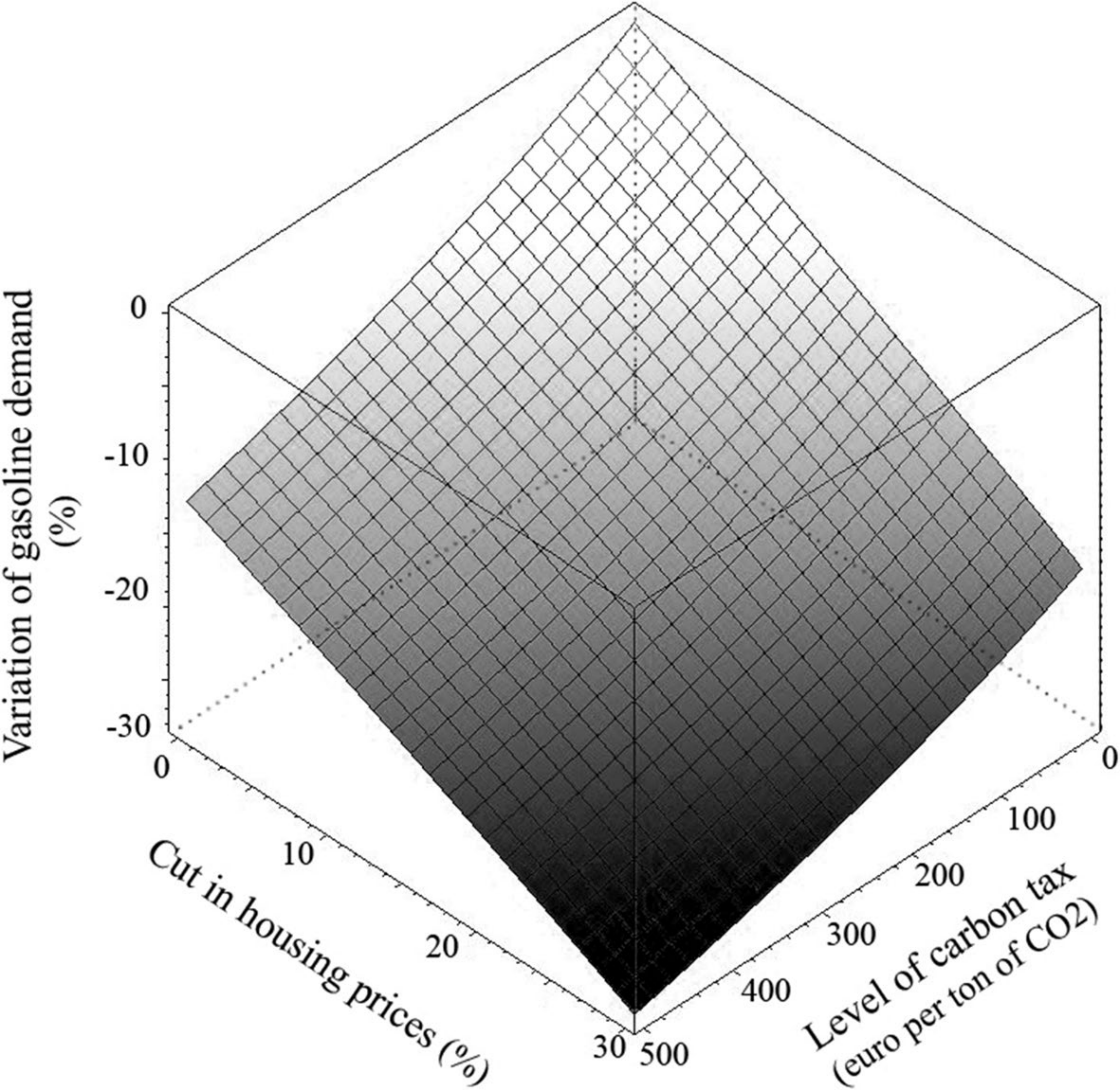
Variations in fuel demand (vertical axis) according to levels of carbon tax and decrease in housing prices. Modelling results obtained with data for Paris. Source: Lampin et al. (2013)

Reducing emissions from transport in cities and their suburban environs through traditional climate policy instruments (e.g. through a carbon tax) is more difficult when inner-urban neighbourhoods are less accessible and less affordable. Exclusionary mechanisms act as a countervailing force to the rising transportation costs induced by the climate policy, pushing households outwards rather than inwards. Said differently, the costs of mitigating intracity transportation emissions are higher when inner-urban housing prices are higher. An illustration is provided by Lampin et al. (2013), who in the case of the Paris metropolis estimate how decrease in housing prices and carbon tax can substitute to achieve the same amount of emission reductions (Fig. 1).

Making inner-urban neighbourhoods more accessible and more affordable can provide suburban households with more capacities to relocate in areas with lower commuting distance and thus adjust to transportation costs increase following mitigation policies. This is particularly important for low- and middle-income households, who spend a greater portion of their income on housing and transportation and nonetheless are more likely to be locked into locations that are distant from their jobs. Such policies may also improve social acceptance, in a context where households may feel helpless in front of rising fuel prices, as illustrated by the “yellow vests” protests in 2019.^1^

Making inner-urban neighbourhoods more accessible and more affordable thus has the potential to reduce both the social costs and the economic costs of mitigation policies. This is already a policy objective in its own right in many places, for social, health, and economic reasons, and it is clearly a complex endeavour (Benner and Karner 2016). Climate mitigation provides an additional rationale to support this objective, even though it is beyond conventional climate policy. In fact, potential synergies exist. For example, revenues derived from climate policies could provide additional resources to support such programmes, and indeed some climate policy initiatives already include provisions to use their revenues towards low-income groups. For example, Karner and Marcantonio (2018) discuss California State Law SB 535, which requires 25% of the revenues of the California cap-and-trade programme to be spent on GHG-reducing investment that benefit disadvantaged communities. Such broader approaches might be necessary in order to build sufficient support for climate action, and Bergquist et al. (2020) find higher support for climate policy packages in the USA when affordable housing programmes are included. Nonetheless, the mitigation implications of keeping inner cities more accessible and affordable for low- and middle-income households often remain out of or is only emerging in the debates surrounding the planning of fast-developing cities in many developing countries (Grant 2015; IADB 2012; Khosla and Bhardwaj 2019).

### Transformational change in transport systems in Costa Rica

Costa Rica communicated its National Decarbonisation Plan (NDP) to the United Nations Framework Convention on Climate Change (UNFCCC) in December 2019, setting a goal of zero-net GHG emissions by 2050. The NDP starts from a vision of a decarbonised economy by 2050 and works backward to identify sectoral pathways to be met in 2035 and policy packages to put the economy on track towards the long-term goal (Waisman et al. 2019). The NDP can be understood as back-casting from visions for transformation, which were agreed in a process involving key stakeholders undertaken by the government (Government of Costa Rica 2019).

The largest reductions in net emissions are set to occur in the transport sector, presently responsible for 51% of the country’s carbon dioxide emissions, which would see a 7.4 MtCO2e reduction by 2050 (Godínez-Zamora et al. 2020; Groves et al. 2020). While the overall plans refer to “net zero emissions” and uses CO2-eq, in the case of transport, the main challenges are to reach net zero CO_2_ emissions — as elaborated in the decarbonization plan for the transport sector (Government of Costa Rica 2019), which is based on three pillars and a set of goals:

- Pillar 1: Mobility system based on safe, efficient, and renewable public transport and on active and shared mobility schemes. By 2035, 70% of buses and taxis would be zero emissions, and the TRP will operate 100% electric. By 2050, 100% of buses and taxis will be zero emissions, an increase of at least 10% in nonmotorised travel within major urban areas.
- Pillar 2: Transformation of the fleet of light vehicles to zero emissions, fueled by renewable energy. By 2035, 25% of the fleet will be electric; by 2050, 100% of new light vehicle sales will be zero-emission vehicles. By 2050, 60% of the light vehicle fleet will be zero emissions.
- Pillar 3: Promotion of freight transport that adopts zero or the lowest possible energy sources, technologies, and modalities. By 2050, at least half of freight transport will be highly efficient and will have reduced emissions by 20% compared to 2018 emissions (Government of Costa Rica 2019).

The three pillars integrate approaches to land use and urban planning; efficient, affordable, safe, and comfortable public transport; and behavioural changes towards local activities and remote activities (Bataille et al. 2020). Thus, transformational change can be seen to be cross-cutting, across several sectors, systems, and policy instruments.

Importantly, the plan includes policy packages and enablers that extend beyond conventional climate policy. While it provides guidelines about the technological and market changes that are often associated with climate policy, the plan also introduces institutional, fiscal, regulatory, and social changes to enhance these technological changes (Government of Costa Rica 2019). Informed by perspectives from government actors, private sector, civil society, and academia, a participatory approach helped define the transformational narratives to be communicated in a common language (Godínez-Zamora et al. 2020). These enablers were found to be drivers of transformational change, in our analysis of the example of SDPS in Costa Rica. Just transition measures are also established, encouraging people, communities, and companies to reorient or adapt their activities.

The NDP 2018–2050 is the basis for developing the National Development and Public Investment Plan (2018–2022). The near-term plan details the actions that the current administration will implement; some may yield results immediately, while other changes may take longer. The NDP will also inform the updating and formulation of new sector policies and the country’s public investment system (Government of Costa Rica 2019).

### Shifts to provide universal energy access and control illegal deforestation in Brazil

In the past two decades, Brazil has arguably shifted its development pathway to reduce GHG emissions while also making progress towards several sustainable development goals. However, the case of Brazil is also an example illustrating that such shifts may be reversed. Under previous administrations (between 2003 and 2016), Brazil implemented a sequence of policies across multiple sectors. These policy packages simultaneously increased minimum wages of low-income families, achieved universal energy access, improved education levels and the public health system, and raised quality of life and well-being for most of the population (Da Silveira Bezerra et al. 2017; Grottera et al. 2017, 2018; La Rovere et al. 2018). This led to significant social benefits, reduction of income inequality, and poverty eradication (Da Silveira Bezerra et al. 2017; Grottera et al. 2017), reflected in a decrease of the Gini coefficient and rise of the human development index (Grottera et al. 2018; La Rovere 2017).

Between 2003 and 2016, regulatory policies by the federal government were effective in controlling illegal deforestation and expansion of cropland farming into native ecosystems, while social incentives improve quality of life of local communities (Bustamante et al. 2018; Nunes et al. 2017; Soterroni et al. 2018, 2019). The Ministry of Environment used regulatory instruments to limit deforestation rates, together with implemented economic instruments that provided benefits to those protecting local ecosystems and enhancing land-based carbon sinks (Hein et al. 2018; Nepstad et al. 2013; Nunes et al. 2017; Simonet et al. 2019; Sunderlin et al. 2014; van der Hoff et al. 2015). The private sector, aligned with public policies and the civil society, implemented the Amazon Soy Moratorium, a voluntary agreement that bans trading of soybeans from cropland associated with cleared Amazon rainforest and blacklists farmers using slave (Heilmayr et al. 2020). Although deforestation driven by soybean cropland, livestock, illegal logging, and other activities still persisted, the country halved its land-based GHG emissions and reduced deforestation by 78 per cent, between 2005 and 2012 (INPE 2019a, b). This example shows that development delivering well-being can be accompanied by significant mitigation. A long-term and strategic vision was important in guiding enabling policies and mechanisms which extended well beyond emission-specific climate policy.

In more recent years, these shifts in Brazil’s development pathways were reversed. The federal government’s political changes redefined development priorities, favouring short-term vested interests, to the detriment of successful climate mitigation and social development policies. The current administration has not funded mandates of environmental agencies and weakened forestry protection laws, notably the Forest Code, while approving the expansion of cropland to protected Amazon rainforest areas and being permissive to illegal land grabbers (Ferrante and Fearnside 2019; Rochedo et al. 2018). As a result, in 2021, Brazil reported the highest deforestation rate in this decade, a loss of over 13,000 km^2^ of native rainforest, and a twofold increase compared to previous decade (INPE 2022). Leading scientists and institutions express concern that the Amazon may reach a tipping point in the near future, potentially irreversible, making it impossible to remediate the rainforest and lost ecosystems, and restore carbon sinks and indigenous people knowledge (Assis et al. 2020; INPE 2019b; Lovejoy and Nobre 2018; Nobre 2019). Furthermore, recent studies suggest that the Amazon biome is no longer a carbon sink, mainly due to anthropogenic interference in the forest carbon cycle (Maia et al. 2020; Rammig and Lapola 2021).

The challenge to mitigation due to rising deforestation rates is exacerbated by a shift in Brazil’s energy matrix. Fossil fuel subsidies and other fiscal benefits to increase exploitation of domestic oil resources may create carbon lock-ins that inhibit further low-carbon investments (Lefèvre et al. 2018). However, according to Rochedo et al. (2018), mitigation costs in the energy sector in Brazil are three times higher than reducing deforestation and increasing land-based carbon sinks. Consequently, Brazil may struggle to realise its contributions to the Paris Agreement.

The example of Brazil’s policies to improve social development and simultaneously reduce its GHG emissions shows that it is possible to shift development pathways towards sustainability by redirecting policies and instruments that promote economic growth and social inclusion and at the same time control illegal deforestation. However, it also reveals that policies are not always sustained and can be reversed, when federal government and governmental agencies waive environmental protection laws and economic instruments.

### Mitigation and preserving equality in Sweden

Between 1990 and 2018, Sweden’s territorial emissions declined at an annual rate of 2.3% (with an acceleration of this rate after 2000), which may be compared to a global emissions growth rate of 1.5% (SEPA 2020a, b). During the same period, living standards improved, and inequality remained relatively low (albeit increasing, from a Gini coefficient of 24.8 in 1992 to 28.8 in 2017), while, under the UNDP Human Development Index, Sweden’s global ranking improved slightly, from 9th to 8th (UNDP 2019; World Bank 2020). What enablers and levers were important in Sweden’s development pathways to shift broadly towards increased sustainability in ways that simultaneously reduced emissions?

Sweden’s emissions decline, and high level of human development has been generated by a wide range of policies, similar to those of other high-income countries but with its specific features. Emissions have for many years been discouraged by high taxes on fossil energy. In 1991, the country was among the first with a carbon tax, introduced as part of green tax shift that reduced personal income and corporate tax rates (Jonsson et al. 2020: p 2). In 2020, this tax was the highest in the world (US $126 per ton) and had contributed to a significant reduction in emissions while still only amounting to 1.0% of total tax revenues (Andersson 2019; Jonsson et al. 2020; Sweden 2020). The carbon tax is complemented by the EU Emissions Trading System (ETS), which covers much of Sweden’s industry, exempting it from the domestic carbon tax (Hållö 2020: 17).

With regard to human development, Sweden’s high level is associated with a high per-capita income and a comprehensive system of social transfers and services. The latter, which are funded and mainly provided by the public sector, include education (free at all levels) and health care (with limited and capped patient payments) (Swedish Institute 2020a, b). The high level of human development underpins high global rankings in terms of innovation and economic complexity and facilitates adaptation to changing economic conditions (Ferrarini and Scaramozzino 2016). Such non-climate efforts have made the introduction of policies that reduces emissions more feasible and politically palatable.

However, to reach the 2045 net zero GHG emissions goal, annual emission cuts will almost have to be tripled compared to the 2000–2018 average. This will require technological progress that raises energy efficiency and the price competitiveness of nonfossil energy for transportation and industry. In the policy area, major changes may include more uniform pricing of GHG emissions via the carbon tax and the EU ETS, complemented by an EU carbon border tax (Andersson 2019; Flam 2019; Hållö 2020; Wetterstrand 2020).

To address broader goals related to equality, income growth, and employment, including in emerging green sectors, such a transformation will require careful calibration of fiscal policy. Given that they eventually will decline, carbon tax revenues, it may be preferable to use carbon tax revenues to finance investments and temporary transformative programmes, which also may be designed to budget neutral, especially important in the wake of Covid-19.^2^

For Sweden, a key enabling condition for the development pathway shift has been the political support that the vision of a low-carbon society enjoys, strong governance, and an adaptable economy. The political support is evidenced in the approval of the Paris Agreement and decarbonisation from all parliamentary parties except one — the Sweden Democrats. Such support may be due to the popular perception that the policies pursued are effective, fair, and not very costly for individuals (Drews and van den Bergh 2016; Konjunkturinstitutet 2019; Zetterberg et al. 2022) Strong governance facilitates the pursuit of policy packages that are effective and fair also in reality. It is likely that, thanks to strong economic adaptability capacity to innovate, the population thinks that that progress towards a zero-carbon society is possible with limited short-run costs and long-run gains, especially if the transformation is successful at the global level. Being a fossil-fuel importer makes the shifts much easier, both politically and economically.

The energy price hikes of 2022 after the outbreak of the Russia-Ukraine war have not lead to any questioning of existing climate targets. However, they have highlighted the fact that such price hikes are socially unacceptable, and that current short-run responses, which may subsidise fossil fuel (capping its prices below market levels), may be at odds with long-term targets and fail to draw on Sweden’s relatively strong capacity to use alternative policies that promise to be more effective both in terms of mitigation and inequality reduction (Zetterberg et al. 2022; Researchers’ Desk 2022).

### Coal transition led by a state-owned enterprise in India

Given the imperatives of climate action, a just transition away from coal will be necessary in India. Accelerating mitigation is urgent, and India’s ambitions for renewable energy are high. However, deeper transformational change transitions are best considered on decadal time-scales (Chandra 2018). India’s energy economy relies extensively on coal — about 202 of 377 GW of total installed capacity for electricity generation in 2020 (MoP 2020). About 151 GW of new coal power and three-quarters subcritical was added since 2006, while in the same period, inefficient and polluting plants were closed. There are significant risks of assets in coal mining and coal-fired electricity generation being stranded — estimated at US $100 billion (Vishwanathan et al. 2018).

A key institution for a coal transition is a public sector undertaking (PSU), Coal India. Chandra (2018) argues that “within state capitalist systems, PSUs have considerable room for endogenous change; external conditionality and mandates are much more likely to succeed when PSUs themselves have the capacity, resources, and leverage to pursue such agendas”. Chandra documents the considerable political influence of the Coal India, with the ability to advocate for rule changes.

Innovation would be needed on technology — modelling of scenarios that contribute to keeping global temperature well below 2 °C assumes “synchronised stringent actions which include: maximising the efficiency of the existing coal fleet …; scaling up new and alternative fuels (renewables and storage, nuclear, gas); reducing end-use energy demands; developing a coherent strategy for the future energy systems to manage risks and avoid stranded assets; and boosting innovation and commercialisation of CCUS” (Vishwanathan et al. 2018). Coal India is committed to invest US $763 million by March 2024 to build 14 solar projects to decarbonise its processes, help power its mining operations, and cut costs (Garg and Vishwanathan 2021). Chandra (2018) points to the ability of PSUs or SOEs^3^ in state capitalist systems to drive institutional innovations, arguing that “adaptive SOEs create operational, financial and political space for themselves in the face of evolving external environments”. He documents how Coal India has built up various adaptive characteristics over four decades of existence, working within the India political and economic system, and thereby creating potential for endogenous change. The challenge of low emissions energy development is a challenge which will require innovation of SOEs as developmental actors, building on existing operational capacity and resource self-sufficiency (Chandra 2018). It will also require shifts of finance — both international and domestic — from high-emission coal into low- and zero-emitting technologies. The investments required up to 2030 in the power sectors have been estimated at US $2 trillion (Vishwanathan et al. 2018).

Taken together, these challenges suggest that policy packages across several domains well beyond conventional climate policy are necessary for a coal transition in India: institutional arrangements, finance, technology, and behaviour. Gupta and Garg (2020) explore a “development first” (DEVF) scenario for transitions in transport in India, which prioritises development objectives — farmer incomes, employment, and reducing dependence on oil imports. Soft-linking an economy-wide model (IMACLIM-IND) with a bottom-up techno-economic model (AIM/Enduse), they back-cast against long-term objectives — developmental in DEVF, carbon neutrality (CNT), and “synchronising” both development and climate goals (SYNCH). In DEVF, substitution of oil imports is a key factor, as this can pay for a significant part of the incremental costs of a transport transition. This saving is carried into the most favourable scenario, SYNCH, which “reduces the dependence on crude oil and natural gas imports by 68% for the year 2050 compared to BAU scenario…. [with] cumulative savings of 5.8 trillion USD from 2013 to 2050” (Gupta and Garg 2020). The SYNCH scenario does not reach zero CO_2_ in 2050 but 80% and 67% reduction in carbon emissions from road and rail transport respectively by 2050 compared to BAU (*ibid.*). Energy efficiency almost doubles, and the study finds major co-benefits in reduced air pollution and congestion in cities (Gupta and Garg 2020).

### Change in economic structure in South Africa

How might South Africa achieve its key development imperative of job creation and remain within a constrained carbon budget? In this example, national modelling was used to explore a shift in economic structure in the south (Altieri et al. 2015, 2016). South Africa is a good example, as its historical development pathway has led high emissions and high unemployment. The country’s priority development goals are to address the triple challenges of poverty, inequality, and unemployment (NPC 2011). Jobs are an apex priority, in that reducing unemployment alleviates poverty, and if the employment is among less skilled workers or absorbs unemployed, it may reduce inequality.

To explore this question, Altieri and colleagues (2016) used a linked modelling framework to explore a possible future shift in economic structure. A shift in economic structure here means a reallocation of capital and labour to low emissions and employment-intensive sectors. Much of this shift in development pathways is focused on the emission-intensive energy sector, but the “minerals-energy complex” has an effect on the entire political economy (Burton 2011; Fine and Rustomjee 1996). Analytically, the modelling linked an energy with an economy-wide model, described elsewhere (Merven et al. 2017).

The study started from the current structure of the economy, which is not able to absorb the high levels of unskilled labour. It developed a future scenario named “economic structure”, changing the structure of the economy to enhance low-carbon high-labour absorbing growth and by allowing the economy to increase trade openness while at the same time meeting a 14 Gt CO2-eq cumulative energy sector, over the period 2016–2050 (Altieri et al. 2016).

To implement this scenario in a national modelling framework, Altieri et al. (2016) started by identifying high-employment, low-carbon sectors. In the linked national models, they then represented government prolabour policy, incentives to shift private investment to labour-absorbing activities, and assuming trade openness. More broadly, this would represent a change from capital-intensive development of the past to employment-intensive growth (Black 2016). In technical terms, this involved decreasing the elasticity of substitution between labour and capital, exogenously increasing capital productivity by 50% in the identified sectors over a 35-year simulation, while relaxing trade elasticities and increasing regional trade exports. Keeping GHG emissions in the energy sector within 14 Gt CO2-eq over the same 35-year period was another user-defined constraint.

**Fig. 2:**
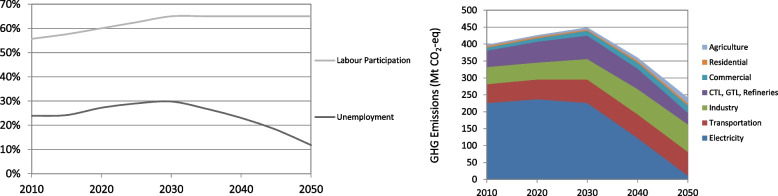
Results from modelling shift in economic structure scenario on reduced unemployment (left panel) and energy-related CO_2_e emissions by sector (right panel). Source: Altieri et al. (2016)

The economic structure scenario thus represents a very different approach to many techno-economic models, which prioritise least-cost solution above all. It simulates a shift to more labour-intensive, low-emissions development. The results reported for economic structure show the structural shifts resulting in an increased uptake of labour into the economy by 2050. “From 2010 to 2050, the model results in the unemployment rate decreasing from 25% to 12%, and the percentage of people living below the poverty line decreasing from 49% to 18%” (Altieri et al. 2016) (see Fig. 2).

Unemployment declines rapidly after 2030, as shown in the left-hand panel in Fig. 1. The right-hand panel shows an overall GHG emissions trajectory that stays within a 14 Gt CO_2_e budget — by design — with a clear shift across sectors, with emissions from electricity nearing zero by 2050.

Models, including the one used in this example, are able to explore future development pathways, thanks to the fact that they focus on problem-relevant parts of reality while abstracting from other parts. Through such an exercise, it is possible to envision futures that reflect shifts from the historical development pathway to ones with lower (and eventually net zero) emissions and increased social justice. Such analysis opens up the conversation to a wide-ranging consideration of the broader policy tools that could help shift SA to an employment intensive and low-emissions development path, achieving climate and other sustainable development objectives (Winkler and Black 2021).

The examples presented in this section have illustrated how actors might shift development pathways in different contexts — some historical cases, others modelling of possible future shifts, some economy wide, and others focused on sectors. With this context-specific information, we turn next to an assessment of the cases across enablers.

## Assessment of examples of SDPS across enablers

From the assessed examples in “Examples of how to shift development pathways”, what can we learn about the roles of different enablers and levers in bringing about an intentional shift towards sustainability, in ways that make more dramatic reductions in emissions possible? What might actors across different contexts learn from examples elsewhere? To explore these questions, we assess the examples across a set of enabling conditions (which we also refer to as enablers, levers and tools).

**Fig. 3:**
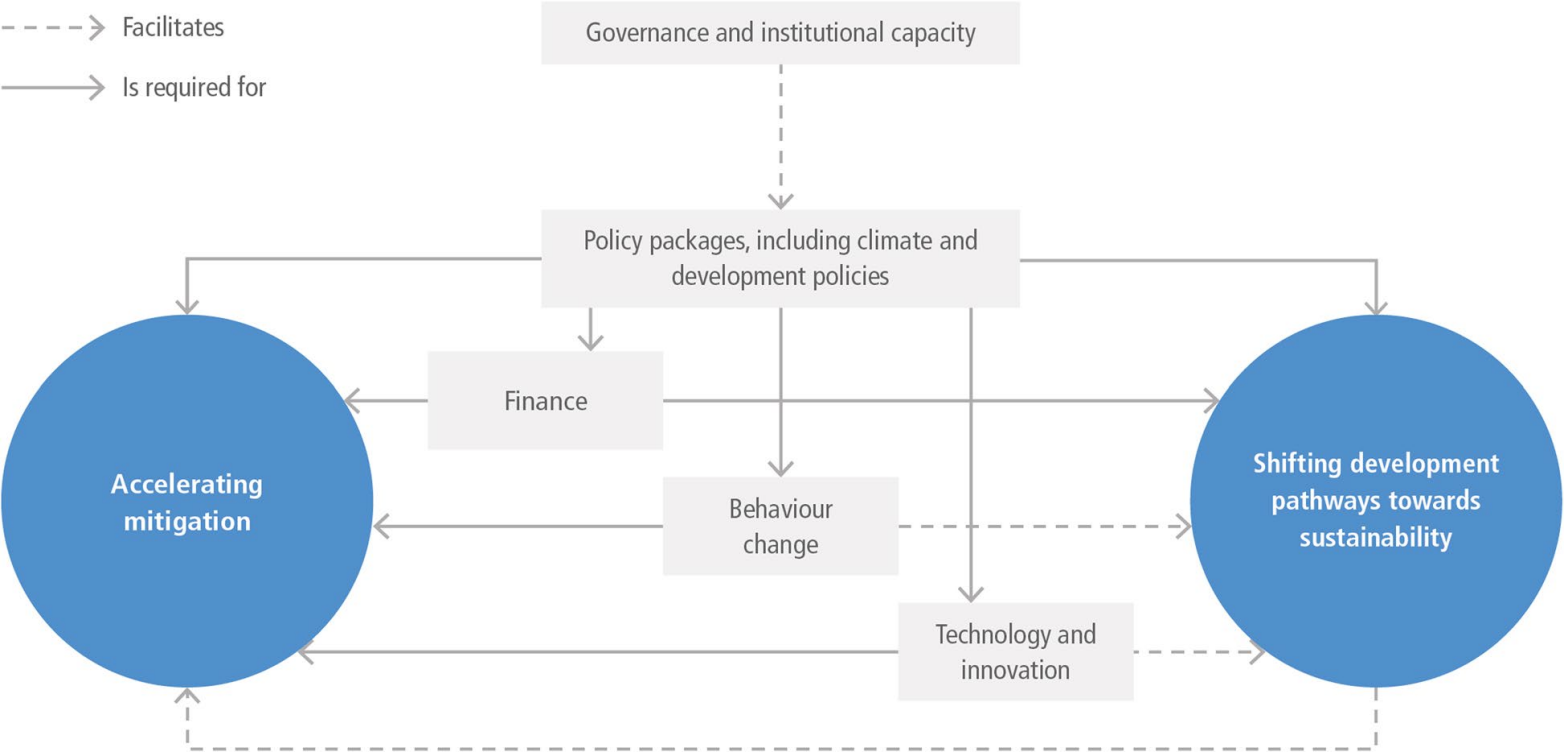
Enabling conditions. Source: Adapted from IPCC chapter 4 Lecocq et al. (2022a) and (IPCC 2018)

The IPCC special report on 1.5 °C (2018) outlined six high-level categories of enabling conditions, applied here to SDPS. Figure 3 shows these enabling conditions — governance and institutional capacity; policy packages – both climate and development-focused policy – finance, behaviour change; and technology and innovation. The dashed arrows in the figure illustrate that these enabling conditions can facilitate both accelerating mitigation and shifting development pathways to increased sustainability. Invariably, SDPS requires some enablers, as suggested by the solid arrows.

We present a comparative analysis of the different examples of SDPS, selected by applying the framework outlined in “Concepts and case study method”, and elaborated in “Examples of how to shift development pathways”, assessed against enabling conditions, Table 2 below. The examples are shown across row headings and the enabling conditions as column headings. The entries in the table provide our assessment of which enabling conditions made change happen in each case. Given the qualitative methods we use, it is possible that other analysts might consider other enablers more important. To make transparent the basis of our assessment, the findings on what enabled a shift for each case are presented in summary in referencing the sub-section above (where citations to the literature can be found). Reading across the rows, we can compare which enabler was significant across several countries. There are some similarities (though the detail of how the enabling condition applied may be different). Comparing similarities and differences provides a comparative analysis on enabling conditions of SDPS. In other words, in this section, we provide an explanation of which enablers are found across *different* cases.

**Table 2:** Assessment of cases of SDPS across enabling conditions

Enablers	Education policy as a driver of sprawl (see Education, family choices and public policy section)	Making inner cities affordable to low- and middle-income households (Making inner-cities affordable and accessible to low- and middle-income households)	Infrastructure planning for decarbonsing transport systems in Costa Rica (Transformational change in transport systems in Costa Rica)	Brazilian shifts to remove emissions and improve well-being (Shifts to provide universal energy access and control illegal deforestation in Brazil)	Shifting development pathway in Sweden (Mitigation and preserving equality in Sweden)	Coal transition by SoE in India (Coal transition led by a state-owned enterprise in India)	Shift in economic structure, South Africa (Change in economic structure in South Africa)
Governance and institutional capacity	Quality of public education relies (in part) on institutional capacity Role of teachers’ unions important in employment conditions, and quality of education. (participation)	Meaningful participation of communities difficult to achieve (Karner and Marcantonio 2018) Ability of different actors to achieve affordability very dependent on institutional context. In some cases (e.g. US cities have very broad roles, while in others, e.g. France, national policies are much more important) Identifying priority areas for job — housing fit requires specific data (Benner and Karner 2016)	Planning decarbonisation requires establishing clear long-term goals, to trace a route and deduced the necessary actions in the short and medium term to achieve the goal Visions for transformation are agreed and detailed with various ministries and key stakeholders Planning decarbonisation requires a clear governance model and cross-cutting measures, including transparency, tax reform, financing strategy, digitization, “just transition” labour, inclusion, human rights, and education Efficient organisation of the transport sector and territorial planning with an integrated governance system The decarbonisation plan is used as the basis for the Costa Rica 2050 Strategic Plan (long-term strategy) Substantive institutional changes are identified to reduce barriers to change	Long-term and strategic vision guided enabling policies and mechanisms (2030) Political changes defined development priorities — and back again Institutional capacity created (e.g. IBAMA, ICMBio) but later weakened	Essential to foster it to make sure that government be able to pursue fair and effective policies with credibility (participation) (vision)	Coal India as an institution built up adaptive capacity over four decades Operational, financial, and political space enables endogenous change, in response to external changes including climate	Vision in National Development Plan (NDP) identified jobs identified as an apex priority (to 2030) National Planning Commission established to produce first NDP in 2011 (institution), to achieve more sustainable development
Behaviour	Family choice of housing location is strongly influenced by access to quality educational Education foundational for sustainable development	Accessibility of inner cities to low- and middle-income has racial component, not just economic component. Attitudes towards segregation are thus critical	Adjust the schedules of provision of public transport services and the efficient and accessible electronic payment Campaigns that promote the use of public transport and intermodality	Deforestation driven by illegal logging, mining and livestock activities was controlled by intensive fiscalisation actions by environmental protection agencies. Reducing deforestation central to sustainable development and climate action Civil society and NGOs conducted aggressive campaigns aiming at raising environmental awareness	Strong behavioural response to carbon pricing is important (as it limits the levels needed to reach emission targets	Coal India engaged in politics, economy, and society Developed ability to advocate for rule changes	Change in economic structure, as modelled) would shift to tertiary and quaternary sectors, well-being created by services and information
Innovation and technology	Educational approaches that improve educational quality. (*Sivan — new teaching methods?)*		Modernise infrastructure and standards Modernise the concession scheme Innovative zero-emission technologies, including hydrogen and fuel cells Innovate and create integration and management capacities for a quality intermodal system	Innovative tools of land use modelling and satellite systems to monitor deforestation (satellite-based real-time detection of deforestation system) R&D investment on land use change and deforestation monitoring	Essential to strive to be able to absorb new technology and preferably be on the frontier, both for domestic mitigation and to develop globally competitive products for export	Institutional innovation — SOEs as developmental actors More efficient, less air polluting coal technologies Shifts to other electricity generating technologies	
Finance	Certain funding choices result in more resource disparities than others	Housing policies require financing, part of which can be derived from the proceeds of climate policies Two elephants in the room: interest rates and the appropriation of land rents. Challenge for more sustainable development	Identify and avoid “lock-in” investments in the short term. Avoid the promotion and adoption of “transitional” transport technologies that create barriers to the decarbonisation of the transport system in the medium and long term The decarbonisation plan is used as the basis for the development of the National Development and Public Investment Plan (2018–2022), together aiming at sustainable development	Conditional agricultural financing schemes designed by the Brazilian Monetary Council (2008) that limits credit access to farms that are noncompliant with the Forest Code	Fiscal policy is essential part of transformation to mobilise resources for needed services and investments, including as a tool for influencing behaviour and income distribution. Fiscal policy can enable more sustainable development	Coal transitions require shifts of finance from high- to low-emissions, for more sustainable energy development pathway Estimated investment requirements US $2 trillion up to 2030 in power sector	Incentives to shift private investment to labour-absorbing activities Reallocation of capital and labour, i.e. assets, to more employment-intensive, low emissions sectors
Policy packages	Size and source of funding of public education is determined by policy decisions Incentives for entering teaching profession and accepting positions in public education		For each axis, policy packages are proposed that combine concrete planning, institutional or regulatory measures, project implementation, access to financing, citizen acceptance, and avoiding lock-in Policy packages not only plan a change but also promote it, finance it, and support it with institutional capacities and the elimination of barriers that may hinder its adoption	Sequence of policy packages, both mitigation and social well-being Use of regulatory and economic instruments Forest Code that defines the target zero illegal deforestation by 2030 Action Plan for the Prevention and Control of Deforestation in the Legal Amazon defines actions across different governmental institutions and proposed procedures for monitoring and environmental control Rural Environmental Registries (CAR in its Portuguese acronym) that obliges landholders with and without formal property rights to declare the size and boundaries of their land holdings Monitoring and registering landholdings in the blacklisted districts that do not comply with the Forest Code Soy Moratorium (2006)	Policy packages, attuned to political realities, often with a fiscal component, are essential for broad human development, to limit inequalities, and to foster an economy that can grow green-economy incomes and jobs	Policy packages across several domains will be needed for a coal transition in India	Pro-labour policy would be key to shift — in reality, not just model — from capital-intensive development of past to employment-intensive growth

Most cells in Table 2 are filled with entries reflecting our assessment of key enablers and levers. Overall, the diversity of levers shown means that one needs multiple enablers. We find no “silver bullet” — one enabling condition that would address all climate action — but rather a wide range of policy efforts that extend far beyond conventional climate policy. The last row of Table 2 shows that policy packages and sequences were important in the seven examples, supporting the argument that transformative shifts in development pathways to fundamentally alter emission pathways require action across a range of policy realms, supporting enablers in multiple domains. With these qualifications, we nevertheless can draw some more general lessons from our comparative analysis of seven cases, following our inductive approach.

Based on our analysis, we find that some enablers are widely applicable, whereas other instances are more context specific. While there is no single enabler or lever or factor that shifted a development pathway, reading down the columns provides an overview of a set of key enablers. We are not suggesting that there are “universal” enabling conditions, limiting our finding to some high-level enabling conditions appear being applicable across several contexts. That said, the enablers shown in these cases already show that countries could learn from what has worked elsewhere.

Some enablers work in all cases examined here — but exactly how still depends on the instruments and context. Finance is always needed, but it is not the same kind of finance. Conditional agricultural finance in Brazil is different to fiscal policy as applied in Sweden. Several of the cases in Table 2 refer to a set of policies, rather than one single instrument. Another enabler is a long-term vision, giving direction to and shaping policy packages. Each case refers to some aspect of sustainable development, understood as local or national objectives, not framed— in these cases — explicitly as achieving the SDGs agreed globally.

The empty cells in Table 2 suggest that not all enabling conditions apply in a particular case, but rather that the enablers that matter vary across the cases.

Some enablers are specific to a case, strongly related to contextual factors. Governance and institutions seem to be more context specific than other rows. Institutions have a particular history in each society and their role in governance in particular. The Brazilian space agency, INPE, plays a particular role in that country. School districts in the USA have particular significance and governance structures.

Participation by a broader set of actors is a theme across the cases, but the sets of actors are quite different: teachers unions are important in US education; inner city communities in Paris. Some institutions have a long history, such as Coal India, and financial institutions in Sweden, whereas the NPC in South Africa is relatively recent, and in Brazil, IBAMA, and ICMBio were specifically created as environmental regulatory agencies. Further research might delve more deeply into the ability of different actors to participate, whether they have the capacity to influence decisions that shift development pathways. “Capacity is not the ability to implement someone else’s agenda but the ability to set and pursue your own agenda and, in that sense, it should be a core element of any development narrative” (Sokona 2021).

One can also see very different kinds of innovation, as a broad enabling condition. In several examples, innovation refers to technologies. However, in the Costa Rican transport system, modernising of the concession schemes was a crucial innovation with links to governance, technology and finance.

Sweden has achieved mitigation while preserving equality, enabled by political support for the vision of a low-carbon society, strong governance and an adaptable economy. A general conclusion from Sweden’s experience is that the conditions that facilitate success in the fight against climate change are similar to the conditions that facilitate success in terms of other development objectives. The Swedish example suggests that the essential ingredients are governance, human and technological development and a strong engagement with the rest of the world. Lessons are not limited to a developed country like Sweden. Costa Rica’s decarbonisation plan combines policies into packages, integrating land use, urban form, transport and behaviour change. Developed through a participatory back-casting process, the NDP informs near-term plans and investments. Brazil shifted development to deliver well-being and mitigation, especially through deforestation, driven by a strategic and adaptative long-term vision. However, recent political changes reversed such shifts.

To implement an “all of society, all of economy” approach to SDPS, countries can draw on the many enablers, as shown in Table 2 as a whole. There are many enablers that have worked in other contexts. How soon such enablers will yield results — in mitigation and other dimensions of sustainable development, deserves, further research. Some enablers may yield results rapidly, whereas others may take time. However, given the urgency of the climate crisis, we argue that putting enablers in place is an urgent priority.

## Conclusion

This paper started with the premise that we need to shift development pathways to accelerate and broaden mitigation. Yet, the concept of successful shifts and transformations need to be made concrete, as we do here through analysis of examples of SDPS and enablers. We have offered lessons from carefully selected examples of how to shift development pathways in different contexts. And we asked how enablers can help achieve shifts in different contexts. We argued that policies beyond conventional climate policy will be necessary and can simultaneously advance other sustainability objectives.

In outline, we outlined our framework for selecting examples in “Concepts and case study method”, provided details of seven examples in “Examples of how to shift development pathways”, and presented a comparative analysis of the examples and their enablers in “Assessment of examples of SDPS across enablers”. Our approach is inductive, and we offer some conclusions from this empirical analysis here. We identify and analyse historical and future, sectoral, and economy-wide examples of shifting development pathways towards sustainability (SDPS): education policy as a driver of sprawl in the USA, making inner cities affordable to low- and middle-income households in Paris, infrastructure planning for decarbonising transport systems in Costa Rica, Brazilian shifts to remove emissions and improve well-being, shifting development pathway in Sweden, coal transition by state-owned enterprise in India, and modelled shifts in economic structure in South Africa. Following our case study method, these examples are not presented as representative, and further research is needed to explore a larger number of cases and deriving lessons across countries and policy areas.

Our approach is to analyse these examples against enablers. Many of the enablers that we identify go beyond the domain of traditional climate policy. The examples include policies related to development priorities, including housing, urban form, education, transport systems, energy access, and poverty alleviation. The examples suggest that links between such domains — e.g. links between education and climate — should be integrated in policy packages. The packages would involve broader group of actors, not only environment ministries. No single enabler was critical in all cases, i.e. there is no “silver bullet”. Rather, multiple enablers and involvement of a broad range of actors (central and local governments, organised labour, private sector, social movements, NGOs) are needed to achieve multiple objectives. We argue that mitigation requires an “all of society, all of economy’ approach”. Accelerating mitigation and shifting development pathways towards sustainability are complements; considering SDPS broadens and deepens mitigation options.

The findings suggest that countries could learn from what has worked elsewhere. However, context matters. Some enablers, like governance and institutions, are context specific. The actors are many and differ by case. Innovations may be crucial in many areas, among them technology, governance, and local finance systems. Other enablers are more widely applicable across several cases, including finance, long-term vision, and focus on sustainable development objectives. Yet how these enablers apply differs, for example whether finance refers to fiscal policy or R&D subsidies.

In sum, putting in place broader enablers is an urgent priority. This is the case, whether the shifts to increased sustainability happen quickly or take decades. Without the broadening of policy options achieved by considering SDPS, we will not achieve deep decarbonisation. By learning lessons from examples of shifting development pathways, we can open up options for faster, deeper, and broader mitigation and sustainable development.

## Data Availability

Qualitative analysis, no data file

## Footnotes

1 The yellow vests movement in France was spurred by increase in fuel prices, consecutive to an increase of the carbon tax rate. While motivations for protests were diverse, perceived inequity in the distribution of the costs of environmental transition was a common thread (Mehleb et al. 2021). In particular, impact of the tax on committed expenditures, notably transport expenditures, has been perceived as an “attack on household budgets” (Blavier 2022).

2 To exemplify, carbon tax revenues may be used to subsidise purchases of high-emitting vehicles for scrapping. Budget-neutral programmes may penalise high-emitting consumption typical of high-income groups and encourage low-emitting consumption typical of low-income groups, in the process generating new behavioural patterns (Hållö 2020: 22-24).

3 Public sector undertakings are the term used in India for state-owned enterprises (SOEs).
